# Effect of Casein Phosphopeptide-amorphous Calcium Phosphate Treatment on Microtensile Bond Strength to Carious Affected Dentin Using Two Adhesive Strategies

**DOI:** 10.5681/joddd.2014.026

**Published:** 2014-09-17

**Authors:** Mahmoud Bahari, Siavash Savadi Oskoee, Soodabeh Kimyai, Firoz Pouralibaba, Farrokh Farhadi, Marouf Norouzi

**Affiliations:** ^1^Dental and Periodontal Research Center, Tabriz Medical Sciences University, Tabriz, Iran; ^2^Assistant Professor, Department of Operative Dentistry, Faculty of Dentistry, Tabriz Medical Sciences University, Tabriz, Iran; ^3^Professor, Department of Operative Dentistry, Faculty of Dentistry, Tabriz Medical Sciences University, Tabriz, Iran; ^4^Assistant Professor, Department of Oral Medicine, Faculty of Dentistry, Tabriz Medical Sciences University, Tabriz, Iran; ^5^Assistant Professor, Department of Oral and Maxillofacial Surgery, Faculty of Dentistry, Tabriz Medical Sciences University, Tabriz, Iran; ^6^Post-graduate Student, Department of Oral and Maxillofacial Radiology, Faculty of Dentistry, Hamadan Medical Sciences University, Hamadan, Iran

**Keywords:** Carious affected dentin, casein phosphopeptide–amorphous calcium phosphate, etch-and-rinse, microtensile bond strength, self-etch

## Abstract

***Background and aims.*** The aim was to evaluate the effect of casein phosphopeptide-amorphous calcium phosphate (CPP-ACP) on microtensile bond strength (μTBS) to carious affected dentin (CAD) using etch-and-rinse and self-etch adhesive systems.

*** Materials and methods.*** The occlusal surface of 32 human molars with moderate occlusal caries was removed. Infected dentin was removed until reaching CAD and the teeth were randomly divided into two groups based on the Single Bond (SB) and Clearfil SE Bond (CSE) adhesive systems. Before composite resin bonding, each group was subdivided into three subgroups of ND, CAD and CPP-ACP-treated CAD (CAD-CPP) based on the dentin substrate. After dissecting samples to l-mm-thick cross-sections (each subgroup: n = 13), μTBS was measured at a strain rate of 0.5 mm/min. Data was analyzed using two-way ANOVA, independent samples t-test and post-hoc Tukey tests (α=0.05).

*** Results.*** Bond strength of both adhesive systems to ND was significantly higher than that to CAD (P <0.001) and CAD/CPP (P < 0.001). There were no significant differences between the μTBS of SB to CAD and CAD-CPP (P > 0.05).μTBS of CSE to CAD-CPP was higher than that to CAD; however, the difference was not significant (P > 0.05). Significant differences were found between SB and CSE systems only with CAD substrate (P < 0.001).

*** Conclusion.*** Regardless of the adhesive system used, surface treatment of CAD with CPP-ACP did not have a significant effect on bond strength. However, bond strength to CAD was higher with SB rather than with CSE.

## Introduction

In modern dentistry, the main purpose of removing carious dentin during cavity preparation for a conservative restoration is to remove solely the external denaturated dentin called infected dentin, which facilitates the protection of internal dentin called carious affected dentin (CAD) and prevents progression of the carious lesion.^1^ After removal of caries and preparation of the cavity for an adhesive restoration, the bulk of the cavity walls consists of CAD; previous studies have shown a low microtensile bond strength (μTBS) to CAD in comparison with intact dentin (ND) using self-etch and etch-and-rinse systems.^2-4^Morphologic changes in CAD are associated with a decrease in μTBS of self-etch and etch-and-rinse systems. The physical and chemical properties of CAD are completely different from those of ND. In the inter-tubular CAD, the high porosity and exposure of collagen fibers along with a decrease in mineral content and the surface energy are seen in comparison to those of ND.^2-4^However, there are no bacteria in the CAD region and despite the attack by organic acids on the mineral and organic content of dentin, collagen cross-linking remains intact; these intact collagen fibers can function as a scaffold for remineralization of intertubular dentin.^5-7^

Recaldent, a milk-based bioactive material, consists of two parts: casein phosphopeptide (CPP) and amorphous calcium phosphate (ACP). CPP is produced of the casein present in milk and has a considerable capacity for the transfer and stabilization of calcium phosphate and as a result, increases calcium and phosphate levels in the dental plaque. ACP is biologically active and can release calcium and phosphate ions in order to maintain supersaturated levels. Calcium and phosphate ions easily diffuse through the porous lesion and are deposited in the partially demineralized crystals, re-forming apatite crystals.^8,9^ This technology has been evaluated in various in vitro and in vivo studies and its role in preventing enamel and dentin demineralization and their remineralization has been substantiated.^9,10^

Adhesive systems implement their effects on enamel and dentin through one of the etch-and-rinse or self-etch mechanisms. Technique sensitivity due to over-etching, over-drying or over-wetting is the most important disadvantage of etch-and-rinse systems.^11^ Self-etch strategy is based on the simultaneous etching and priming of dentin covered by the smear layer through the action of an acidic primer. These systems result in less technique sensitivity, elimination of separate etching and rinsing steps and facilitation of the bonding mechanism.^2^ There is controversy over the efficacy of self-etch adhesive systems. Some studies have shown that their bond strength to ND and CAD is comparable to that of etch-and-rinse systems; however, some others have shown a lower bond strength.^2,3,11-13^

Scholtanus et al^14^ evaluated the microtensile bond strength of three different simplified adhesive systems to caries-affected dentin and reported no significant differences in bond strength values to normal dentin between the three adhesives. Adper Scotchbond 1 XT is a 2-step etch-and-rinse adhesive, and Clearfil S3 Bond is a 1-step self-etching or all-in-one adhesive; they have shown significantly lower bond strength values to caries-affected dentin. For Clearfil SE Bond a 2-step self-etching adhesive, bond strength values to normal and caries-affected dentin were not significantly different.^14^

Adebayo et al^15^ evaluated microshear bond strength of two self-etching/priming adhesives (Clearfil SE Bond [CSE] and G-Bond [GB]) to normal dentin following application of a CPP-ACP and the effect of smear layer removal before paste application and preconditioning. CPP-ACP application did not reduce MSBS for CSE but significantly reduced MSBS for GB when the smear layer was removed before paste application. Preconditioning did not improve or worsen dentin MSBS for CSE or GB with/without CPP-ACP, except when polyacrylic acid conditioning was used with GB.^15^

Low bond strength to tooth structure can lead to early microleakage around restoration margins which can cause marginal staining, hypersensivity, recurrent caries, and pulpal irritation.^16,17^ Since one of the reasons for low bond strength to carious affected dentin is a decrease in its mineral content and exposure of collagen fibers^4^ and CPP-ACP can remineralize dentin, and because to date no study has evaluated the effect of CPP-ACP pretreatment on bond strength to CAD, this study aimed to evaluate the effect of surface treatment with CPP-ACP on µTBS to CAD and compare it with those of ND with the use of etch -and-rinse and self-etch adhesive systems. The null hypothesis was: there are no significant differences between µTBS to ND, CAD and CAD-CPP, regardless of the adhesive system used.

## Materials and Methods

### Collection and Initial Preparation of Teeth

Human molars with moderate occlusal surface caries, which had been extracted for periodontal reasons, were selected for the purpose of the present study. Radiographic exposures were used to determine dentinal extension of carious lesions; 32 teeth in which the coronal caries on the radiogram had affected almost half of the dentin thickness were selected. All the debris and remaining soft tissue tags were removed from tooth surfaces using a universal scaler and all the teeth were stored in 1% chloramine T solution at 4°C for a month before the study.

### Preparation of Dentinal Substrate

Initially, the enamel and dentin of the occlusal surface were removed using a diamond saw (D&Z, Lemgo, Germany) in a low-speed straight handpiece (NSK, Tochigi, Japan) under continuous water spray to achieve a flat surface. The tooth surface was stained with Pele Tim foam pellets impregnated with a caries-disclosing agent (Caries Marker, VOCO, Cuxhaven, Germany). After 10 seconds the tooth surfaces were rinsed for 10 seconds.^18,19^ The infected dentin was stained red, which was removed with a #4 round carbide bur (SS White, Lakewood, NJ, USA) and a sharp excavator. The application was repeated until no additional dentin coloration occurred. Then, a dental explorer was used to examine the remaining dentin in order to ensure the removal of all the infected dentin and reach CAD. Then 600-grit silicon carbide paper (Phoenix Beta, Buehler, Germany) was used under running water to achieve a standard flat surface for the bonding procedure. CAD was marked on the remaining tooth structure on the buccal, lingual, mesial or distal areas using a marker. The prepared specimens were divided into two groups of 16 based on the adhesive system used.

### Treatment Groups

*Bonding with the Etch-and-rinse Adhesive System:* In 8 of 16 selected specimens for bonding with the etch-and-rinse adhesive system, bonding of composite resin to the prepared tooth surface was performed without application of CPP-ACP using Single Bond (SB) (3M ESPE, St. Paul, MN, USA) adhesive system. After application of the adhesive and light-curing according to manufacturer’s instructions, Valux Plus composite resin (3M ESPE) was placed on the tooth surface in three 1.5-mm-thick increments, which added up to a total of 4.5 mm. Each layer was cured for 20 seconds using Litex 680A light-curing unit (Dentamerica, 18320 Bedford Circle, City of Industry, CA 91744, USA) at a light intensity of 600 mW/cm^2^. In 8 remaining specimens the bonding procedure was performed after application of CPP-ACP. To this end, the paste containing CPP-ACP (GC Tooth Mousse, GC EUROP N.V., Interleuvenlaan 13, B3001, Leuven) was placed on the prepared tooth surface containing CAD and sound dentin for 5 consecutive days, 15 minutes daily. Then composite bonding procedure was carried out similar to the previous 8 samples and the specimens were placed in distilled water at 37°C for 24 hours in an incubator.

Subsequently, the teeth were mounted on a plastic plate using cyanoacrylate glue (Mad Wolf, Akpinar Yapi Melzemeleri, LTD, Istanbul, Turkey) and cut bucco-lingually and mesiodistally using a diamond blade at a moderate speed using a cutting device (Thin Sectioning Machine Inc, Rochester, NY, USA) under water spray, perpendicular to the bonded surface, to produce rods with approximate dimensions of 1 mm. Each tooth specimen yielded 1-2 sound dentin fragment(s) and the same number of CAD fragments. In this way, 13 sound dentin samples, 13 CAD samples and 13 CAD samples treated with CPP-ACP (CAD-CPP) were achieved. Sound dentin samples were only selected from the teeth which had not been treated with CPP-ACP.

*Bonding with the Self-etch Adhesive System:* Remaining 16 selected molars were used to evaluate bond strength of self-etch adhesive system to ND, CAD, and CAD-CPP in the same method as that used with etch-and-rinse adhesive system except for the fact that bonding to dentin was carried out with Clearfil SE Bond (CSE) (Kurraray Medical Inc., Okayama, Japan) according to manufacturer’s instructions. All the other steps were the same as previously described.

In order to reduce selection bias, random allocation of samples into study groups was performed using Randlist software version 1.2. Furthermore, the examiners were blinded to the groups of study. The chemical compositions and particulars of study materials are presented in Table 1.

**Table 1:** Study materials, their composition, manufacturer and batch numbers

Materials	Composition	Application Procedures	Manufacturer	Lot Number
Single Bond	2-HEMA, Bis-GMA, Di-methacrylates, Amins, MethacrylaTE Functional co-polymer of polyaacrylic and polyitaconic acid, ethanol, water, photoinitiator	Scotchbond ^TM^ Etchant was applied to the substrate for 15 seconds; Rinsed for 10 seconds and excess water was blotted using a cotton pellet. No air drying was used. Immediately after blotting, 2-3 consecutive coats of adhesive were applied for 15 seconds with gentle agitation using a fully saturated applicator. Gently air thinned for 5 seconds to evaporate solvent. Then, Light-cured for 10 seconds.	3M ESPE, St. Paul, MN, USA	N202333
Clearfil SE Bond	SE Primer: N,N-Diethanol-p-toluidine, MDP, HEMAhydrophilic dimethacrylate, DL-camphorquinone, water. SE Bond: N,N-Diethanol-p-toluidine, MDP, Bis-GMA, HEMA, hydrophobic dimethacrylate, DL camphorquinone, silanated,colloidal silica	Primer: Primer was applied to the substrate with a disposable brush tip, Leaved in place for 20 seconds. Then, the volatile ingredients were evaporated with a mild oil-free air stream. Bond: bond was applied to the entire surface of substrate with a disposable brush tip. After application, the bond film was made as uniform as possible using a gentle oil-free air stream.hen light-cured for 10 seconds.	Kuraray Medical Inc., Okayama, Japan	Primer: 01027A Bond: 01531A
Caries Marker	Propane-1,2-diol resin mixture with coloring agents	First, the softened and clearly discolored dentine was removed. The cavity was wetted with Caries Marker for 5 - 10 s with Pele Tim foam pellets and then rinsed out with water spray. Carious dentine was brightened red. Dyed dentine areas were removed completely. the application was repeated until no additional dentine coloration occurs, being careful to conserve the undyed dentine areas.	VOCO GmbH, 27457 Cuxhaven, Germany	1031066
GC Tooth Mousse	Pure water, Glycerol, CPP-ACP, D-Sorbitol, Silicon dioxide, CMC-Na, Propylene glycol, Titanium dioxide, Xylitol, Phosphoric acid, Guar gum, Zinc oxide, Sodium saccharin, Ethyl-p-hydroxybenzoate, Butyl-p-hydroxybenzoate, Propyl-p hydroxybenzoate	The paste was placed on the prepared tooth surface for 15 consecutive days, 15 minutes daily.	GC EUROPE N.V., Interleuvenlaan 13,B-3001 Leuven	090409s

### µTBS Test

Dimensions of each sample at the interface were measured with a digital caliper (Mitutyo, Tokyo, Japan) and recorded so that interface surface area could be calculated. Then each sample was mounted on the special jig of the Miccrotensile Tester (Bisco, Schaumburg, USA) using cyanoacrylate glue (Mad Wolf, Akpinar Yapi Melzemeleri, LTD, Istanbul, Turkey) and subjected to a tensile strength at a strain rate of 0.5 mm/min until fracture. The force at failure was recorded and the µTBS of each sample was calculated in MPa through dividing the maximum force (Newton) by interface surface area (mm^2^).

### Determining Failure Modes

Subsequently, failure modes were determined under a stereomicroscope (Nikon, SMZ-800, Osaka, Japan) at ×40 magnification:

- Type A: Cohesive failure in dentin
- Type B: Adhesive failure
- Type C: Mixed failure (when more than 25% of Type A was present)
- Type D: Cohesive failure in composite resin

### Statistical Analysis

Normal distribution of data was confirmed by Kolmogorov-Smirnov test (P > 0.05). Then data was analyzed by two-way ANOVA and post hoc Turkey tests using SPSS 15 statistical software. Independent samples t-test was used for the two-by-two comparisons of bond strengths of different substrates between the two SB and CSE adhesive systems. Statistical significance was defined at P < 0.05.

## Results

Means, standard deviations, and standard deviation errors of µTBS values for both adhesive systems and their subgroups are presented in Table 2. The results of two-way ANOVA showed that the effects of both variables (the type of the substrate and the adhesive systems) on bond strength were significant (P < 0.001). In addition, the cumulative effect of the two variables was significant (P < 0.001). Two-by-two comparisons of different substrates with post-hoc Tukey test showed that the differences between the µTBS values of ND/CAD (P < 0.001) and ND/CAD-CPP (P < 0.001) were significant. However, no significant differences were observed between the µTBS values of CAD and CAD-CPP (P > 0.05).

**Table 2:** Means of microtensile bond strength valus (MPa), standard deviations (SD), standard deviation errors (std. error) and failure modes (%)

Groups		Bond Strength (MPa)				Type of Failure Modes (%)			
Adhesive Type	Dentin type	N	Mean	SD	Std.Error	A	B	C	D
	ND	13	30.39	4.84	1.34	(7)	(23)	(32)	(38)
	CAD	13	25.10	4.77	1.37	(0)	(42)	(33)	(25)
SB	CAD-CPP	13	21.99	3.16	0.87	(0)	(50)	(25)	(25)
	ND	13	28.12	3.88	1.12	(7)	(23)	(32)	(38)
	CAD	13	16.76	4.38	1.21	(0)	(61)	(32)	(7)
CSE	CAD-CPP	13	19.38	3.54	1.02	(0)	(50)	(17)	(33)
SB: Single Bond, CSE: Clearfil SE Bond, N: Number, SD: Standard Deviation, ND: Normal Dentin, CAD: Carious Affected Dentin, CAD-CPP: Carious Affected Dentin Treated with Casein Phosphopeptide-Amorphous Calcium Phosphate.									
A: Cohesive failure in dentin, B: Adhesive failure, C: Mixed failure (when more than 25% of Type A was present), D: Cohesive failure in composite resin									

In addition, two-by-two comparisons of the adhesive systems with post hoc Tukey test revealed statistically significant differences in bond strength values between SB and CSE (P < 0.001).

Furthermore, two-by-two comparisons of the different subgroups of each adhesive system with post-hoc Tukey test showed statistically significant differences between µTBS values of SB to ND compared to CAD (P < 0.001) and CAD-CPP (P < 0.001). However, there were no significant differences between the µTBS values of SB to CAD and CAD-CPP (P > 0.05).

There were significant differences between the µTBS values of CSE to ND and CAD (P < 0.001) and CAD-CPP (P < 0.001). µTBS of CSE to CAD-CPP was higher than that to CAD; however, the difference was not significant (P > 0.05).

Furthermore, two-by-two comparison of bond strength values of different dentin substrates between the SB and CSE adhesive systems with independent t-test showed significant differences between SB and CSE systems only with CAD substrate (P < 0.001), with no significant differences from ND and CAD-CPP (P > 0.05).

As seen in Table 2, Type A failure modes were only seen with ND. Type B and Type D failure modes increased and decreased in CAD with CSE and SB bonding systems, respectively.

## Discussion

In recent years, rapid development of the concepts and materials of adhesive dentistry has shifted the strategy of restoration of carious lesions toward the use of conservative methods in cavity preparation and increasing use of tooth-colored restorations. In preparation of cavities for conservative adhesive restoration, the bulk of the cavity walls consist of CAD.^11^ However, bond strength to CAD is lower than that to ND due to a decrease in its mineral content and exposure of collagen fibers.^4^Recaldent technology, which has recently been introduced, has the capacity to remineralize CAD.^8,9^ The present study evaluated the effect of surface treatment with CPP-ACP on µTBS to CAD and compare it with those of ND with the use of etch-and-rinse and self-etch adhesive systems.

Infected dentin and CAD are differentiated based on visual inspection, examination with a dental explorer and staining methods.^18,19^ According to Fusayama et al,^18^ the use of caries-disclosing agents is an acceptable clinical tool for diagnosis and removal of CAD.^18^ At present, due to the carcinogenic effects of fuchsine, propylene glycol and 1% red acid are used to this end, which differentiates between the two carious dentin layers in same manner as fuchsine.^19^ In the present study, all three methods (visual inspection, examination with a dental explorer, and staining methods) were used to increase the accuracy of assessments in the use of the right substrate for bonding.

The results of the present study showed that the bond strength of resin depends on the substrate and the type of the adhesive system, which is consistent with the results of previous studies.^1,2,11^,^20,21^ Furthermore, the µTBS of SB to ND was significantly higher than those of SB to CAD and CAD-CPP; however, the difference between µTBS of SB to CAD and CAD-CPP was not statistically significant.

SB is used along with 35% phosphoric acid to remove the smear layer and demineralize the dentin surface, which opens the dentinal tubules, exposes the collagen fibers and completely evacuates hydroxyapatite crystals.^22^ Insolubility of peritubular dentin results in the formation of funnel-shaped resin tags after infiltration of resin monomers into the dentinal tubules. SB forms resin tags which have a large number of tiny microtags as lateral branches form the main resin tag. These resin tags and infiltration of resin microtags into the lateral branches of dentinal tubules and anastomoses between them might contribute to an increase in bond strength.^15^ In addition, the monomer of this resin can easily penetrate into demineralized dentin and surface collagen bundles to form a strong bond.^23^

Intermittent demineralization and remineralization of dentin in the carious process usually results in the closure of the orifices of dentinal tubules with mineral crystals at CAD. This prevents deep penetration of etching acid and lack of deep infiltration of resin tags into the tubules, too.^11^ SEM observations have shown that the typical hybrid layer and resin tags cannot form in CAD and penetration of resin might be prevented by closure of dentinal tubules with mineral particles.^24^ The hybrid layer is thicker in CAD; however, based on previous studies, a thick hybrid layer cannot increase the bond strength.^25^ The relationship between the length of the resin tags and the bond strength depends on the location of bond in dentin (superficial, intermediate, or deep), dentin permeability (depending on the level of wettability) and orientation of dentinal tubules (parallel with the surface or reticular).^21^ Due to structural properties of CAD, resin tags are shorter and more irregular with less lateral infiltration of resins into the lateral ramifications of dentinal tubules.^24^

Yushiyama et al^20^ reported that the bond strength of SB to ND is significantly higher than that to CAD. In contrast, Xuan et al^11^ showed higher bond strength of SB to ND compared to that with CAD, with no significant differences.

According to Sattabanasuk et al,^26^ surface treatment of ND with CPP-ACP and then etching with phosphoric acid exhibits a residue layer on the surface and a decrease in the length of resin tags with lateral infiltration of resins into the lateral ramifications of dentinal tubules and their bonding with ND, which was attributed to mineralization and seal of the tubules by crystals and prevention of resin infiltration; therefore, it was postulated that application of CPP-ACP on CAD surface might affect bond strength of some adhesive systems such as CSE which have the ability of chemical bonding to dentin. The functional monomer of CSE (10-MDP) can establish an ionic bond with hydroxyapatite crystals.^11,26^ Meanwhile, the CPP-ACP increases deposition of these crystals on dentin surfaces.^27^

Another finding of the present study was the fact that the bond strength of CSE to ND was much higher than those to CAD and CAD-CPP. Moreover, surface treatment of CAD with CPP-ACP resulted in an increase in bond strength of CSE but this increase was not statistically significant. Several studies have shown a higher bond strength of CSE to ND than that to CAD.^2,11,21^ Consistent with the results of the present study, when the primer of CSE is applied to the surface of ND, dentin is partially demineralized to a depth of 1 µm. The formed resin tags are thin and cylindrical with some lateral infiltration into the lateral extensions of dentinal tubules, which seem to be sufficient to produce a strong mechanical retention during the bonding procedure.^1^ Furthermore, the functional 10-MDP monomer, which is an ingredient of CSE, can form a chemical bond with the remaining surface hydroxyapatite crystals, resulting in an increase in bond strength.^26^ However, demineralization phase of carious dentin and the subsequent remineralization cycles usually result in the closure of the orifices of dentinal tubules with mineral crystals and as a result, in less penetration of resin into the tubules compared with ND.^28^ Lack of penetration of CSE adhesive into the tubules to form resin tags might be attributed to intertubular mineral deposits, which are resistant to acids, and to the low acidity of CSE (pH=2) and as a result to less penetration of primer into CAD.^2,29^

Interestingly, based on the results of the present study, it appears that pretreatment of CAD with CPP-ACP might increase the bond strength of CSE to CAD. This increase was not statistically significant. The orifices of tubules are closed by impermeable calcium phosphate in CAD.^2^ CPP-ACP increases deposition of these crystals.^27^ CSE chemically bonds to the deposited phosphate groups by CPP-ACP, resulting in higher bond strength. The functional monomer of CSE, referred to as 10-MDP, can establish an ionic bond with hydroxyapatite crystals.^11,26^

Another important finding of the present study was the fact that the bond strength of SB to CAD was higher than that of CSE; however, in relation to ND and CAD-CPP the two adhesive systems did not exhibit any significant differences, which might be attributed to a better infiltration of resin monomers as a result of the use of 35% phosphoric acid with SB^13^ or the presence of polyalkenoic acid copolymer in its chemical composition.^30^ However, the primer of CSE might not optimally etch CAD and might not be able to sufficiently penetrate into the closed dentinal tubules. There are fewer soluble calcium phosphate crystals in carious dentin. Therefore, a stronger acidity might be necessary to dissolve the crystals of the mineral phase of CAD.^2^

In the present study, cohesive failure modes in dentin were only observed in ND, which might be attributed to the high bond strength values of both adhesive systems to ND compared with those to CAD and CAD-CPP. In the CSE-CAD subgroup, the number of adhesive and cohesive failures in composite resin was higher and lower, respectively, compared to CAD-SB subgroup, which might be attributed to its lower bond strength compared to SB.

Some of the limitations of the present study were the impossibility of long-term evaluation of bond strength and simulation of the oral cavity conditions. Therefore, it is suggested that further long-term studies be carried out in clinical situations to achieve more accurate results. In addition, given the effect of surface treatment of CAD with CPP-ACP on an increase in bond strength of CSE adhesive system, it is suggested that further studies be carried out in this regard with the use of other self-etch adhesive systems so that the results can be extended to clinical situations.

## Conclusion

Under the limitation of the present study it can be concluded that:

1) microtensile bond strength to ND was higher than that to CAD.

2) microtensile bond strength to SB to CAD was higher compared to CSE.

## References

[1] Nakajima M, Ogata M, Okuda M, Tagami J, Sano H, Pashley DH (1999). Bonding to caries-affected dentin using self-etching primers. Am J Dent.

[2] Ceballos L, Camejo DG, Victoria Fuentes M, Osorio R, Toledano M, Carvalho RM (2003). Microtensile bond strength of total-etch and self-etching adhesives to caries-affected dentine. J Dent.

[3] Giriyappa RH, Chandra BS (2008). Comparative evaluation of self-etching primers with fourth and fifth generation dentin-bonding systems on carious and normal dentin substrates: An in vitro shear bond strength analysis. J Conserv Dent.

[4] Zanchia CH, D'Avila OP, Rodrigues-Junior SA, Burnett LH Jr, Demarco FF, Pinto MB (2010). Effect of additional acid etching on bond strength and structural reliability of adhesive systems applied to caries-affected dentin. J Adhes Dent.

[5] Ogawa K, Yamashita Y, Ichijo T, Fusayama T (1983). The ultrastructure and hardness of the transparent layer of human carious dentin. J Dent Res.

[6] Deyhle H, Bunk O, Müller B (2011). Nanostructure of healthy and caries-affected human teeth. Nanomedicine.

[7] Kawasaki K, Ruben J, Tsuda H, Huysmans MC, Takagi O (2000). Relationship between mineral distributions in dentine lesions and subsequent remineralization in vitro. Caries Res.

[8] Andersson A, Sköld-Larsson K, Hallgren A, Petersson LG, Twetman S (2007). Effect of a dental cream containing amorphous cream phosphate complexes on white spot lesion regression assessed by laser fluorescence. Oral Health Prevent Dent.

[9] Kumar VL, Itthagarun A, King NM (2008). The effect of casein phosphopeptide-amorphous calcium phosphate on remineralization of artificial caries-like lesions: an in vitro study. Aust Dent J.

[10] White JM, Eakle WS (2000). Rationale and treatment approach in minimally invasive dentistry. J Am Dent Assoc.

[11] Xuan W, Hou BX, Lü YL (2010). Bond strength of different adhesives to normal and caries-affected dentins. Chin Med J (Engl).

[12] Nakajima M, Sano H, Burrow MF, Tagami J, Yoshiyama M, Ebisu S (1995). Tensile bond strength and SEM evaluation of caries-affected dentin using dentin adhesives. J Dent Res.

[13] Nakajima M, Sano H, Urabe I, Tagami J, Pashley DH (2000). Bond strengths of single-bottle dentin adhesives to caries-affected dentin. Oper Dent.

[14] Scholtanus JD, Purwanta K, Dogan N, Kleverlaan CJ, Feilzer AJ (2010). Microtensile bond strength of three simplified adhesive systems to caries-affected dentin. J Adhes Dent.

[15] Adebayo OA, Burrow MF, Tyas MJ (2008). Dentine bonding after CPP-ACP paste treatment with and without conditioning. J Dent.

[16] Vinay S, Shivanna V (2010). Comparative evaluation of microleakage of fifth, sixth, and seventh generation dentin bonding agents: An in vitro study. J Conserv Dent.

[17] Usha H, Kumari A, Mehta D, Kaiwar A, Jain N (2011). Comparing microleakage and layering methods of silorane-based resin composite in class V cavities using confocal microscopy: An in vitro study. J Conserv Dent.

[18] Fusayama T (1979). Two layers of carious dentin;diagnosis and treatment. Oper Dent.

[19] Fusayama T, Yamada T, Inokoshi S (1993). The use of a caries detector dye during cavity preparation. Br Dent J.

[20] Van Meerbeek B, De Munck J, Yoshida Y, Inoue S, Vargas M, Vijay P (2003). Buonocore memorial lectureAdhesion to enamel and dentin: current status and future challenges. Oper Dent.

[21] Chappell RP, Cobb CM, Spencer P, Eick JD (1994). Dentinal tubule anastomosis: a potential factor in adhesive bonding?. J Prosthet Dent.

[22] Carvalho RM, Mendonça JS, Santiago SL, Silveira RR, Garcia FC, Tay FR (2003). Effects of HEMA/solvent combinations on bond strength to dentin. J Dent Res.

[23] Prati C, Chersoni S, Mongiorgi R, Pashley DH (1998). Resin-infiltrated dentin layer formation of new bonding systems. Oper Dent.

[24] Yazici AR, Akca T, Ozgünaltay G, Dayangaç B (2004). Bond strength of a self-etching adhesive system to caries-affected dentin. Oper Dent.

[25] Yoshiyama M, Tay FR, Doi J, Nishitani Y, Yamada T, Itou K (2002). Bonding of self-etch and total-etch adhesives to carious dentin. J Dent Res.

[26] Sattabanasuk V,  BurrowMF  BurrowMF, Shimad Y, Tagami J (2009). Resin Bonding to Dentine After Casein Phosphopeptide-Amorphous Calcium Phosphate (CPP-ACP) Treatments. J Adhes Sci and Technol.

[27] Adebayo OA, Burrow MF, Tyas MJ (2010). Resin-dentine interfacial morphology following CPP-ACP treatment. J Dent.

[28] Tay FR, Pashley DH, Hiraishi N, Imazato S, Rueggeberg FA, Salz U (2005). Tubular occlusion prevents water-treeing and through-and-through fluid movement in a single-bottle, one-step self-etch adhesive model. J Dent Res.

[29] Azarpazhooh A, Limeback H (2008). Clinical efficacy of casein derivatives: a systematic review of the literature. J Am Dent Assoc.

[30] Van Meerbeek B, Conn LJ Jr, Duke ES, Eick JD, Robinson SJ, Guerrero D (1996). Correlative transmission electron microscopy examination of nondemineralized and demineralized resin-dentin interfaces formed by two dentin adhesive systems. J Dent Res.

